# Advances in Melanoma: From Genetic Insights to Therapeutic Innovations

**DOI:** 10.3390/biomedicines12081851

**Published:** 2024-08-14

**Authors:** Fernando Valdez-Salazar, Luis A. Jiménez-Del Rio, Jorge R. Padilla-Gutiérrez, Yeminia Valle, José F. Muñoz-Valle, Emmanuel Valdés-Alvarado

**Affiliations:** Centro Universitario de Ciencias de la Salud, Instituto de Investigación en Ciencias Biomédicas (IICB), Universidad de Guadalajara, Guadalajara 44340, Mexico; fernando.valdez9882@alumnos.udg.mx (F.V.-S.);

**Keywords:** melanoma, genetic mutations, MAPK pathway, PI3K/Akt/mTOR pathway, epigenetic modifications, tumor microenvironment, immunotherapy, immune checkpoint inhibitors, mRNA vaccines

## Abstract

Advances in melanoma research have unveiled critical insights into its genetic and molecular landscape, leading to significant therapeutic innovations. This review explores the intricate interplay between genetic alterations, such as mutations in *BRAF*, *NRAS*, and *KIT*, and melanoma pathogenesis. The MAPK and PI3K/Akt/mTOR signaling pathways are highlighted for their roles in tumor growth and resistance mechanisms. Additionally, this review delves into the impact of epigenetic modifications, including DNA methylation and histone changes, on melanoma progression. The tumor microenvironment, characterized by immune cells, stromal cells, and soluble factors, plays a pivotal role in modulating tumor behavior and treatment responses. Emerging technologies like single-cell sequencing, CRISPR-Cas9, and AI-driven diagnostics are transforming melanoma research, offering precise and personalized approaches to treatment. Immunotherapy, particularly immune checkpoint inhibitors and personalized mRNA vaccines, has revolutionized melanoma therapy by enhancing the body’s immune response. Despite these advances, resistance mechanisms remain a challenge, underscoring the need for combined therapies and ongoing research to achieve durable therapeutic responses. This comprehensive overview aims to highlight the current state of melanoma research and the transformative impacts of these advancements on clinical practice.

## 1. Introduction

Melanoma is a malignant neoplasm originating from melanocytes, presenting a significant challenge in oncology due to its aggressive nature and tendency to develop metastasis [1]. Despite medical advances in early detection and treatment, melanoma remains a major cause of morbidity and mortality worldwide [2]. According to the latest GLOBOCAN report in 2022, melanoma resulted in 331,722 new cases and 58,667 deaths [3]. However, it is important to note that these figures may be under-reported due to a lack of comprehensive epidemiological surveillance in many regions. Factors such as limited access to healthcare, diagnostic discrepancies, and inadequate reporting systems contribute to the under-representation of the true incidence and mortality rates of melanoma in the general population. In recent years, the advent of genomic technologies has revolutionized our understanding of melanoma pathogenesis by uncovering the intricate interplay between genetic alterations and disease progression [4]. This review aims to provide a comprehensive overview of recent advances in melanoma research, focusing on genetic insights and therapeutic innovations. By examining the latest data on molecular pathways, epigenetic modifications, and emerging treatments, we seek to highlight the significant progress and ongoing challenges in the fight against this aggressive skin cancer. Through an integrative analysis of the latest research findings and clinical advancements, we strive to provide a comprehensive overview of the genetic basis of melanoma and its transformative impact on oncological practice.

First and foremost, various risk factors associated with melanoma development can be broadly classified into environmental and genetic factors [5]. Prolonged and unprotected exposure to ultraviolet (UV) radiation is the primary environmental risk factor associated with the disease [6]. There are three different types of UV radiation that vary based on their wavelengths: UVA (340–400 nm), UVB (280–320 nm), and UVC (200–280 nm). UVA is mainly used in tanning beds and has a greater ability to penetrate the dermis of the skin, although it is less genotoxic than UVB radiation. UVB radiation is strongly associated with DNA damage, as it can form thymine dimers that compromise the stability and integrity of genetic material [5,7,8].

In addition to environmental factors, genetic predisposition plays a crucial role in melanoma susceptibility. Familial clustering of melanoma cases has long been recognized, highlighting the importance of genetic factors in the disease’s etiology [9]. Several high-penetrance melanoma susceptibility genes, including *CDKN2A* and *CDK4*, are associated with familial melanoma syndromes characterized by an autosomal dominant inheritance pattern [10]. These genes encode proteins involved in cell cycle regulation and tumor suppression. Germline mutations in *CDKN2A* and *CDK4* can significantly elevate an individual’s lifetime risk of developing melanoma. Notably, genome-wide association studies (GWAS) have identified 85 susceptibility loci, highlighting the complex genetic landscape of melanoma and the substantial roles of these genetic factors in its development. Many of these genetic variants are in or near genes involved in pigmentation pathways, DNA repair mechanisms, and immune responses [11,12,13]. These findings underscore the genetic complexity of melanoma susceptibility and the need for continued research to elucidate the underlying mechanisms leading to disease development.

## 2. Molecular Mechanisms

Melanoma is characterized by the complex interplay of molecular events that drive tumor initiation, progression, and metastasis [14,15]. Understanding the underlying molecular mechanisms governing melanoma pathogenesis is crucial for developing effective therapeutic strategies and improving patient outcomes.

### 2.1. MAPK Signaling Pathway

**Figure 1:**
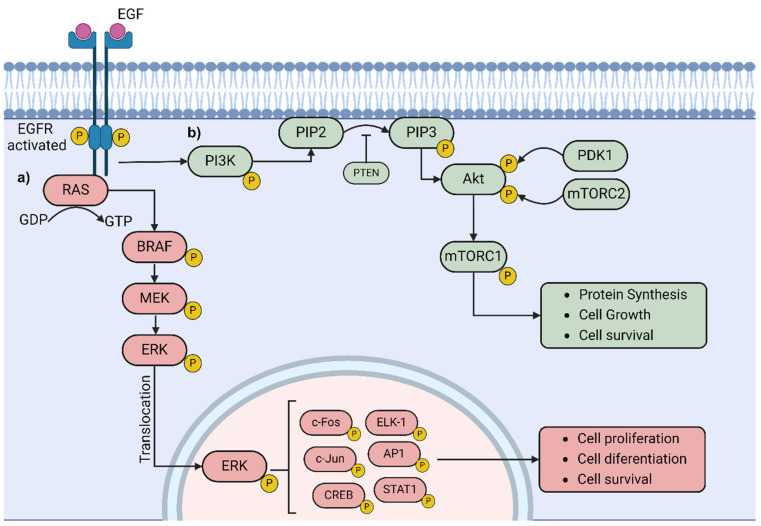
MAPK/MEK/ERK and PI3K/Akt/mTOR Signaling Pathways. (**a**) The binding of epidermal growth factor (EGF) to its receptor (EGFR) on the cell membrane leads to the activation of Ras by facilitating the exchange of GDP for GTP. Activated Ras (Ras-GTP) interacts with and activates BRAF, which, in turn, phosphorylates and activates MEK. Subsequently, MEK phosphorylates and activates ERK. Phosphorylated ERK translocates to the nucleus, where it interacts with transcription factors such as c-Fos, c-Jun, ELK-1, AP1, CREB, and STAT1, causing changes in gene expression that promote cell proliferation, differentiation, and survival. (**b**) The same activated EGFR recruits and activates PI3K. Activated PI3K converts PIP2 (phosphatidylinositol 4,5-bisphosphate) into PIP3 (phosphatidylinositol 3,4,5-trisphosphate) at the inner leaflet of the plasma membrane; this reaction can be regulated by PTEN, which dephosphorylates PIP3. PIP3 recruits Akt to the membrane, where it is phosphorylated and activated by PDK1 (3-phosphoinositide-dependent protein kinase-1) and mTORC2 (mechanistic target of rapamycin complex 2). Activated Akt leads to the activation of mTORC1. Activated mTORC1 promotes protein synthesis, cell growth, and survival. Created with BioRender.com.

The MAPK signaling pathway, as shown in Figure 1, is a central regulator of cellular processes such as proliferation, survival, and differentiation, and its dysregulation is implicated in melanoma pathogenesis [16,17]. Mutations in *BRAF*, especially the V600E mutation, are the most common genetic alterations in melanoma, leading to the constitutive activation of BRAF kinase activity and sustained activation of downstream effectors like MEK and ERK [18]. Similarly, mutations in *NRAS* result in aberrant activation of the MAPK pathway, promoting melanoma cell proliferation and survival [19].

Studies have elucidated the molecular mechanisms underlying MAPK pathway activation in melanoma and its role in tumor progression [20]. Besides genetic alterations, the dysregulation of upstream signaling molecules and feedback mechanisms can further amplify MAPK signaling in melanoma cells [21,22]. For example, increased expression of receptor tyrosine kinases (RTKs) like c-Met and EGFR can promote ligand-independent MAPK pathway activation, contributing to tumor growth and metastasis [23].

Targeting the dysregulated MAPK signaling pathway has emerged as a promising therapeutic strategy in melanoma [24]. BRAF inhibitors, such as vemurafenib and dabrafenib, selectively target mutant BRAF proteins and have shown significant clinical efficacy in patients with BRAF V600 mutations. Combined therapies targeting multiple nodes of the MAPK pathway, such as BRAF and MEK inhibitors, have demonstrated improved response rates and prolonged progression-free survival compared to single-agent therapy [25,26].

The MAPK signaling pathway intersects with many other pathways that play diverse and crucial molecular roles. One such related pathway is the PI3K/Akt/mTOR pathway. The following section will delve into the PI3K/Akt/mTOR signaling pathway and its implications in melanoma biology.

### 2.2. PI3K/Akt/mTOR Signaling Pathway

The PI3K/Akt/mTOR pathway is a crucial signaling cascade involved in various aspects of melanoma biology, including tumor growth, survival, metabolism, and therapeutic resistance [27,28]. The dysregulation of this pathway is frequently observed in melanoma and contributes to disease progression [29].

The activation and regulation of the PI3K/Akt/mTOR pathway: Like the MAPK signaling pathway, the PI3K/Akt/mTOR pathway is activated in response to extracellular signals, such as growth factors and cytokines, through the activation of RTKs and other cell surface receptors [30,31]. PI3K activation leads to the production of phosphatidylinositol 3,4,5-trisphosphate (PIP3), which recruits and activates protein kinase B (Akt) at the plasma membrane [32]. Activated Akt phosphorylates downstream effectors, including mTORC1, regulating various cellular processes [33,34].

The dysregulation of PI3K/Akt/mTOR signaling is often observed in melanoma due to genetic alterations, such as mutations and amplifications in pathway components [35,36]. This dysregulation confers resistance to conventional therapies and targeted inhibitors. Consequently, targeting the PI3K/Akt/mTOR pathway has emerged as a promising therapeutic strategy for melanoma treatment. Recent studies have focused on the development of novel inhibitors targeting key components of the PI3K/AKT/mTOR pathway. One such inhibitor, KTC1101, a pan-PI3K inhibitor, has shown promising results in recent studies. In preclinical models, KTC1101 demonstrated significant tumor growth inhibition, with a reduction in tumor size by 65% compared to controls. Moreover, KTC1101 has been shown to modulate the tumor microenvironment by reducing the number of immunosuppressive myeloid-derived suppressor cells (MDSCs) and regulatory T cells (Tregs) within the tumor. This modulation is crucial because it alleviates the immunosuppressive effects that often hinder the effectiveness of immune responses against the tumor. Furthermore, when combined with anti-PD-1 therapy, KTC1101 enhanced the antitumor immune response, leading to a 75% reduction in tumor growth. These findings suggest that KTC1101 not only targets tumor cell proliferation but also positively influences the tumor microenvironment by promoting a more favorable immune landscape for immune-mediated tumor suppression. Ongoing clinical trials are further evaluating the effectiveness of KTC1101 in combination with other therapies to overcome resistance and improve patient prognosis [37,38].

While the PI3K/AKT/MTOR pathway plays a pivotal role in melanoma cell survival and proliferation, another critical aspect of melanoma progression is the deregulation of the cell cycle. Understanding how cell cycle checkpoints are disrupted provides further insight into the mechanisms driving melanoma growth and offers additional targets for therapeutic intervention.

### 2.3. Cell Cycle Dysregulation

Cell cycle dysregulation is a hallmark of melanoma, characterized by uncontrolled tumor proliferation and growth. At the heart of this dysregulation are alterations in the finely orchestrated machinery that governs cell cycle progression [39]. The aberrant expression and activity of key regulatory proteins, such as cyclins and cyclin-dependent kinases (CDKs), drive cells through cell cycle checkpoints [40]. This includes anomalies in the G1/S transition phase, where cyclin D1 and CDK4/6 push cells past the G1 checkpoint, bypassing regulatory mechanisms that normally prevent damaged cells from entering the replication phase [41]. Moreover, melanoma cells often exhibit alterations in checkpoint control mechanisms, including the loss of function of critical tumor suppressor proteins such as p16 and p53 [42,43]. The disruption of these checkpoints allows cells with genomic instability or DNA damage to evade arrest and continue through the cell cycle, fueling tumor progression [44].

For example, recent studies have explored the impact of BCR-ABL tyrosine kinase inhibitors, such as nilotinib and AT-9283, on cell cycle regulation in human melanoma A375P cells. These inhibitors significantly reduce cell proliferation and migration, with nilotinib and AT-9283 showing 40–60% reductions in cell proliferation rates and 30–50% decreases in cell migration in treated A375P cells. Specifically, nilotinib and AT-9283 impede the G1/S transition of the cell cycle. This effect is achieved through the downregulation of cell cycle-associated proteins, including cyclin E, cyclin A, and CDK2, leading to a 50–70% reduction in RB phosphorylation levels. Consequently, the expression of E2F target genes, such as *CCNA2*, *CCNE1*, *POLA1*, and *TK-1*, is suppressed by approximately 40–60% in nilotinib and AT-9283-treated cells. These findings highlight the potential of these inhibitors to control cell cycle progression in melanoma by targeting the RB-E2F pathway, thereby reducing the proliferative capacity of melanoma cells and potentially improving therapeutic outcomes [45].

Another point of interest is the significant potential of CDK4/6 inhibitors in melanoma treatment. Targato et al. (2024) reviewed the clinical application and molecular mechanisms of CDK4/6 inhibitors, emphasizing their role in melanoma therapy. The review discusses the efficacy of CDK4/6 inhibitors, such as palbociclib, ribociclib, and abemaciclib, which have shown promising results in clinical trials. These inhibitors work by targeting the CDK4/6 pathway, which is frequently altered in melanoma, leading to uncontrolled cell proliferation. Key findings from Targato et al. include a 50–70% reduction in tumor size in clinical trials involving melanoma patients. Additionally, the review highlights the potential of combining CDK4/6 inhibitors with other treatments, such as BRAF/MEK inhibitors and immunotherapies, to enhance therapeutic outcomes and overcome resistance mechanisms. These inhibitors function by downregulating cyclin D1 and CDK4/6 activity, leading to the hypophosphorylation of RB1 and subsequent inhibition of E2F-mediated transcription, which is crucial for cell cycle progression [46].

In a study by Wang et al. (2023), the focus was on the development and efficacy of new CDK4/6 inhibitors and PROTACs targeting CDK4/6. The study presents promising preclinical data showing that these inhibitors can significantly reduce tumor growth in melanoma models. These molecules have shown the ability to selectively degrade CDK4/6, providing a novel and effective way to inhibit these kinases. This approach has the potential to overcome the limitations of traditional inhibitors, such as drug resistance and off-target effects. The study presents data from recent preclinical studies showing that PROTACs can effectively reduce CDK4/6 levels, leading to significant tumor growth inhibition. Clinical trials are underway to evaluate their efficacy in melanoma patients [47].

On the other hand, a study conducted by Vanderbilt University Medical Center explored how MDM2 influences resistance to CDK4/6 inhibitors in melanoma treatment. This study revealed that targeting MDM2 in combination with CDK4/6 inhibitors could enhance the therapeutic efficacy and overcome resistance in melanoma cells. The findings suggest a potential combinatorial approach to improve patient outcomes [48]. The inclusion of CDK4/6 inhibitors and PROTACs targeting CDK4/6 in melanoma treatment regimens represents a significant advancement in the management of this aggressive cancer. By targeting critical pathways involved in cell cycle regulation, these therapies offer a promising approach to control melanoma progression and improve patient outcomes. Ongoing research and clinical trials will further elucidate the full potential of these therapeutic strategies.

Following the disruption of cell cycle regulation, epigenetic modifications also play a significant role in melanoma progression. These changes, which affect gene expression without altering the DNA sequence, contribute to the complex landscape of tumor development and resistance. Understanding these modifications provides additional avenues for therapeutic strategies.

### 2.4. Epigenetic Modifications

Epigenetic modifications play a crucial role in gene expression regulation without altering the DNA sequence itself. These modifications, including DNA methylation, histone modifications, and non-coding RNAs, are fundamental for controlling cellular processes like differentiation, proliferation, and apoptosis. In melanoma, epigenetic dysregulation significantly contributes to tumor initiation, progression, and therapy resistance.

#### 2.4.1. DNA Methylation

**Figure 2:**
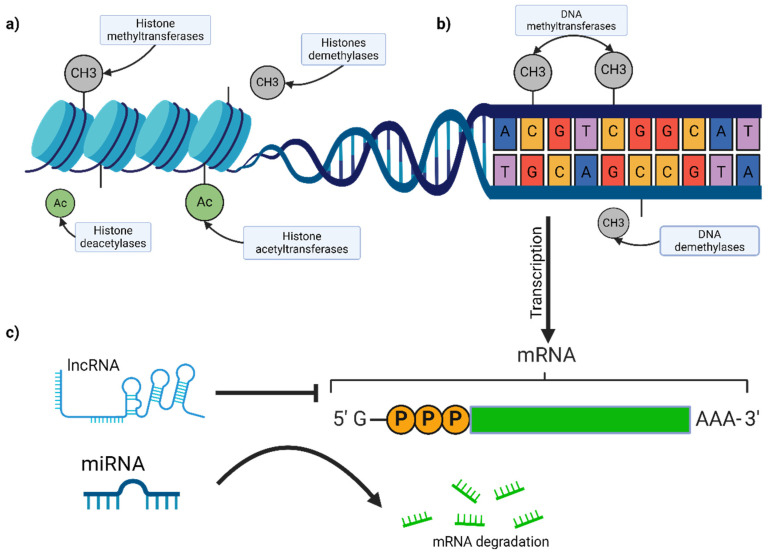
Epigenetic Modifications. The diagram shows some of the epigenetic modifications that occur in the genome as part of gene expression regulation. (**a**) Histone Modifications: methylations and demethylations carried out by histone methyltransferases and histone demethylases, respectively, and acetylations and deacetylations conducted by histone acetyltransferases and histone deacetylases, respectively. (**b**) DNA Methylation: DNA methyltransferases and DNA demethylases can add methyl groups to or remove from cytosines in DNA (particularly in CpG islands) to silence or activate gene expression. (**c**) Non-Coding RNAs: the regulation of mRNAs mediated by lncRNAs and miRNAs, which can bind complementarily to mRNA and lead to its inhibition or degradation. Created with BioRender.com.

DNA methylation, the mechanism shown in Figure 2, involves adding a methyl group to the 5-carbon of cytosine residues, primarily in CpG dinucleotides, leading to gene silencing [49]. Aberrant DNA methylation patterns are a hallmark of melanoma. The hypermethylation of tumor suppressor genes like p16INK4a, RASSF1A, and PTEN results in their silencing, contributing to uncontrolled cell proliferation and survival [50,51,52]. Conversely, global hypomethylation can lead to genomic instability and oncogene activation. For instance, global DNA hypomethylation is associated with constitutive PD-L1 expression in melanoma cells, inhibiting T-cell effector function and enabling immune evasion [53]. Advances in whole genome methylation profiling have identified numerous differentially methylated regions (DMRs) in melanoma, offering potential biomarkers for diagnosis and prognosis [54].

Recent studies have shown that DNA methylation signatures can predict responses to immune checkpoint inhibitors (ICIs) in metastatic melanoma. One study analyzed DNA methylation patterns in two independent cohorts of melanoma patients and developed a machine learning classifier to predict long-term therapy response to ICIs. The study found that specific DNA methylation profiles correlate with better responses to ICIs, suggesting that these profiles could serve as biomarkers for selecting patients who are more likely to benefit from such treatments. The study also highlighted the importance of incorporating DNA methylation profiling into future biomarker research to enhance personalized treatment strategies for melanoma patients [55].

#### 2.4.2. Histone Modifications

Histones, proteins around which DNA is wrapped, undergo various post-translational modifications, such as methylation, acetylation, phosphorylation, and ubiquitination. These modifications can alter chromatin structure and accessibility, regulating gene expression (Figure 2) [56]. In melanoma, the dysregulation of histone-modifying enzymes, such as histone methyltransferases (e.g., EZH2) and histone deacetylases (e.g., HDAC), has been observed [57,58]. Furthermore, recent research has highlighted the interaction between long non-coding RNAs (lncRNAs) and EZH2 as a promising therapeutic target in cutaneous melanoma. EZH2 is a component of polycomb repressive complex 2 (PRC2), which mediates gene silencing through histone methylation. lncRNAs can act as molecular “address codes” for EZH2, directing it to specific genomic loci to exert its repressive function. Targeting the interaction between lncRNAs and EZH2 has shown potential in slowing down melanoma progression [59].

Additionally, recent findings from Moffitt Cancer Center revealed that the HDAC8-mediated inhibition of EP300 drives a transcriptional state that significantly increases melanoma brain metastasis. The study showed that stress-induced HDAC8 activity leads to a neural crest stem cell-like transcriptional state, enhancing melanoma cell invasion and resistance to stress. The study reported that increased HDAC8 activity led to a 50% increase in brain metastasis in a mouse model of melanoma, highlighting the potential of targeting HDAC8 to mitigate melanoma brain metastasis. HDAC8 deacetylates EP300, a histone acetyltransferase, leading to its inactivation and increased melanoma cell invasion. Targeting HDAC8 could, therefore, be a viable strategy to mitigate melanoma brain metastasis [60].

#### 2.4.3. Non-Coding RNAs

Non-coding RNAs, including microRNAs (miRNAs) and long non-coding RNAs (lncRNAs), play critical roles in gene regulation [61]. The dysregulation of ncRNAs contributes to melanoma pathogenesis by acting as either oncogenes or tumor suppressors.

##### MicroRNAs (miRNAs)

Numerous miRNAs are dysregulated in melanoma. For example, the following points must be considered:

miR-211: Typically downregulated in melanoma, miR-211 acts as a tumor suppressor by targeting genes involved in cell migration and invasion, such as NUAK1 and TGFBR2. Its reduced expression is associated with increased melanoma aggressiveness and poor prognosis [62,63].
miR-21: Overexpressed in melanoma, miR-21 promotes tumor growth and metastasis by inhibiting tumor suppressor genes like PTEN and PDCD4, which leads to enhanced cell proliferation and resistance to apoptosis [64,65].
miR-214: Another critical miRNA, miR-214, has been shown to regulate melanoma cell proliferation and survival by targeting transcription factor AP-2 (TFAP2) and promoting the expression of stemness-associated genes [66,67,68].

##### Long Non-Coding RNAs (lncRNAs)

LncRNAs are also pivotal in melanoma progression and metastasis. Notable examples include the following:

MALAT1 (Metastasis-Associated Lung Adenocarcinoma Transcript 1): MALAT1 promotes melanoma metastasis through interactions with chromatin-modifying complexes and the regulation of gene expression involved in cell migration and invasion. It modulates the alternative splicing of pre-mRNAs, influencing cellular processes critical for tumor progression [69,70,71].
BANCR (BRAF-activated non-coding RNA): BANCR is upregulated in melanoma and is involved in cell migration and proliferation. It interacts with the MAPK pathway, further enhancing melanoma cell survival and growth [70,72,73].
Survival-Associated Mitochondrial Melanoma-Specific Oncogenic Non-Coding RNA (SAMMSON): A melanoma-specific lncRNA, SAMMSON, is essential for melanoma cell survival. It interacts with p32, a mitochondrial protein, to regulate mitochondrial function and energy production, highlighting its potential as a therapeutic target [74,75].

#### 2.4.4. Implications for Diagnosis and Treatment

The epigenetic landscape of melanoma offers potential biomarkers for early detection, prognosis, and therapeutic targets. DNA methylation markers, such as p16INK4a hypermethylation, are being explored for non-invasive diagnostic tests [76]. Epigenetic therapies, including DNA methyltransferase inhibitors (e.g., decitabine) and histone deacetylase inhibitors (e.g., vorinostat), are being investigated for their efficacy in melanoma treatment [77,78]. Additionally, targeting dysregulated non-coding RNAs with synthetic oligonucleotides or small molecules holds promise for novel therapeutic interventions [79].

After discussing the impact of epigenetic modifications on melanoma progression, it is important to consider the role of the tumor microenvironment. This dynamic and complex milieu not only facilitates tumor growth and metastasis but also modulates immune responses and influences treatment outcomes. The interactions within this environment are pivotal to understanding melanoma’s behavior and resistance mechanisms.

### 2.5. Tumor Microenvironment

The tumor microenvironment (TME) is a complex entity that plays a fundamental role in melanoma progression and therapeutic resistance [80]. It consists of a diverse array of cellular components, such as immune cells, stromal cells, and endothelial cells, as well as non-cellular elements like the extracellular matrix (ECM) and various soluble factors [81]. These components interact in a highly orchestrated manner, influencing tumor behavior, modulating immune responses, and contributing to the metastatic potential of melanoma cells [82].

#### 2.5.1. Immune Cells

The TME in melanoma is a dynamic ecosystem where multiple immune cells, both from the innate and adaptive immune responses, play critical roles either by combating cancer cells or aiding tumor progression.

##### Tumor-Infiltrating Lymphocytes (TILs)

Tumor-infiltrating lymphocytes (TILs), particularly CD8+ cytotoxic T lymphocytes (CTLs), are crucial for the body’s antitumor response [83]. These cells recognize melanoma cells by detecting specific antigens presented on the surface via class I major histocompatibility complex (MHC) molecules. Once activated, CD8+ TILs can directly destroy melanoma cells, significantly contributing to tumor control [83,84]. However, the efficacy of TILs can be diminished by immunosuppressive cytokines like IL-10 and TGF-β, which are abundant in the TME and can inhibit T-cell function [85,86]. For instance, in the phase II C-144-01 study, the treatment with lifileucel, a commercial, autologous TIL cell product, showed sustained benefits, with long-term survival in at least 20% of patients with advanced melanoma resistant to immune checkpoint inhibitors (ICIs). The study reported 1-, 2-, 3-, and 4-year overall survival (OS) rates of 54.0%, 33.9%, 28.4%, and 21.9%, respectively. The objective response rate (ORR) was 31.4%, with the median duration of response not being reached, indicating durable and ongoing responses in patients. These findings underscore the potential of TIL therapy to significantly improve outcomes in patients with advanced melanoma [87].

##### Macrophages

Macrophages in the TME are versatile and can adopt different phenotypes, playing various beneficial or harmful roles in the body:

M1-like macrophages, “fighter” macrophages, in the immune response produce pro-inflammatory cytokines like IL-12 and TNF-α and generate reactive oxygen species (ROS) that attack tumor cells, promoting antitumor immunity and inhibiting tumor growth [88,89]. M2-like macrophages act more as “healers” for the tumor. They produce anti-inflammatory cytokines like IL-10 and TGF-β and secrete factors like VEGF that promote new blood vessel formation (angiogenesis), aiding tumor growth and survival. M2-like macrophages are usually more prevalent in melanoma and are associated with poor prognosis because they create an immunosuppressive environment that favors tumor progression [90]. In a study analyzing the role of M2-like macrophages in melanoma, it was found that high infiltration of CD206+ M2-like macrophages correlated with a 40% decrease in overall survival compared to patients with low M2-like macrophage infiltration. Additionally, these macrophages were shown to promote immune evasion by inhibiting the activation of CD8+ T cells through the secretion of immunosuppressive cytokines [91].

Recent studies have highlighted the critical role of tumor-associated macrophages (TAMs) in melanoma. TAMs are often polarized towards an M2-like phenotype in the melanoma TME, promoting tumor growth, metastasis, and immune evasion. They secrete various growth factors, cytokines, and enzymes that remodel the extracellular matrix and facilitate tumor invasion. For example, TAMs have been shown to secrete matrix metalloproteinases (MMPs) such as MMP-9, which degrade the extracellular matrix and allow melanoma cells to invade surrounding tissues. In one study, increased levels of MMP-9 were associated with a 50% increase in metastatic potential in melanoma models [92].

Targeting TAMs to reprogram them from a pro-tumorigenic M2-like phenotype to an antitumorigenic M1-like phenotype is a promising therapeutic strategy. Approaches such as using CSF1R inhibitors to deplete M2-like macrophages or using agonists to activate TLRs and promote M1-like polarization are being explored. For instance, a recent preclinical study demonstrated that treatment with a combination of a CD40 agonist and a Dectin-1 agonist significantly increased the number of M1-like macrophages and decreased the number of M2-like macrophages in the TME. This shift in macrophage phenotype was associated with a substantial reduction in tumor growth and improved survival rates in melanoma models [93].

##### Myeloid-Derived Suppressor Cells (MDSCs)

MDSCs are a diverse group of cells that significantly contribute to immunosuppression within the TME [94]. They inhibit T-cell activation through various mechanisms, such as the production of arginase and inducible nitric oxide synthase (iNOS), which deplete nutrients essential for T-cell function and generate ROS that further inhibit T cells [95,96]. Additionally, MDSCs can promote the expansion of regulatory T cells (Tregs), enhancing the overall immunosuppressive environment [97]. Elevated levels of MDSCs in melanoma patients are related to more advanced disease stages and poorer prognosis, as they effectively dampen the immune response against the tumor [98].

Recent studies have shown that high levels of MDSCs correlate with poor prognosis and reduced overall survival in melanoma patients. For instance, a study found that patients with high levels of M-MDSCs had a 30% lower progression-free survival (PFS) rate compared to patients with lower levels of these cells. Additionally, the presence of elevated G-MDSCs was associated with a significant decrease in overall survival rates [96].

In a retrospective study involving advanced melanoma patients treated with anti-PD-1 immunotherapy, blood samples were analyzed for MDSC levels before and during treatment. The study reported that responders to anti-PD-1 therapy had higher levels of M-MDSCs before treatment compared to non-responders (4.1% vs. 3.0%, p = 0.0333). This suggests that MDSC levels could potentially serve as biomarkers for predicting response to immunotherapy [99].

##### Dendritic Cells

Dendritic cells (DCs) are essential for initiating and regulating immune responses. They capture antigens from melanoma cells and present them to T cells, effectively priming the immune system to recognize and attack the tumor [100]. However, in the TME, melanoma cells and other components release factors that impair DC maturation and function. Key factors involved in this immunosuppression include VEGF and prostaglandin E2 (PGE2). These molecules create a hostile environment for DCs, preventing their proper maturation and antigen presentation capabilities, leading to suboptimal T-cell activation and a diminished overall immune response against the tumor [101,102].

#### 2.5.2. Stromal Cells

Stromal cells, including cancer-associated fibroblasts (CAFs), endothelial cells, and adipocytes, play significant roles in the melanoma TME. These cells interact with melanoma cells, influencing tumor growth, invasion, and metastasis [103,104,105].

##### Cancer-Associated Fibroblasts (CAFs)

CAFs are a major component of the TME and contribute to melanoma progression through various mechanisms. They secrete ECM components, growth factors, and cytokines that promote tumor growth and invasion [106]. CAFs produce matrix metalloproteinases (MMPs) that degrade ECM components, facilitating melanoma cell invasion into surrounding tissues [107]. Additionally, CAFs secrete growth factors like TGF-β, IL-6, and hepatocyte growth factor (HGF), which promote tumor proliferation and angiogenesis [108]. CAFs also contribute to immune suppression by recruiting and activating Tregs and MDSCs through the secretion of immunosuppressive cytokines [109].

Targeting CAFs offers a promising therapeutic strategy to inhibit tumor progression and enhance antitumor immunity. Approaches such as using CAR-T cells targeting fibroblast activation protein (FAP) on CAFs, as well as inhibitors of pathways involved in CAF activation and function, are being explored. A recent study demonstrated that targeting the matricellular protein CCN1 in CAFs impaired melanoma metastasis and neovascularization, highlighting its potential as a novel therapeutic target [110].

##### Endothelial Cells

Endothelial cells line blood vessels are crucial for tumor angiogenesis, the process by which new blood vessels form to supply nutrients and oxygen to the tumor. Melanoma cells secrete pro-angiogenic factors such as VEGF, fibroblast growth factor (FGF), and platelet-derived growth factor (PDGF), stimulating endothelial cell proliferation and new blood vessel formation [111]. This vascular network not only supports tumor growth but also provides a pathway for metastatic spread. Targeting angiogenesis, for instance, with VEGF inhibitors can disrupt the tumor blood supply and normalize abnormal tumor vasculature, improving the delivery of chemotherapeutic agents and immune cells to the tumor [112]. 

To address these challenges, combining VEGF inhibitors with other therapeutic agents targeting different pro-angiogenic pathways is being explored. For example, inhibiting sFRP2 (Secreted Frizzled-Related Protein 2) in combination with VEGF can be more effective in older patients. Additionally, therapies targeting endothelial cell signaling pathways and interactions with other cell types in the TME, such as myeloid cells and fibroblasts, are being investigated to enhance the anti-angiogenic response [113].

##### Adipocytes

Adipocytes, or fat cells, in the TME are increasingly recognized for their role in melanoma progression. These cells provide an energy-rich microenvironment by releasing fatty acids through lipolysis, which melanoma cells can use for rapid growth and proliferation [114]. Adipocytes also secrete adipokines like leptin and adiponectin, which can influence melanoma cell signaling pathways involved in tumor growth, inflammation, and metastasis [115]. The interaction between melanoma cells and adipocytes highlights the metabolic symbiosis that can drive tumor progression. Recent studies have shown that adipocyte-derived lipids significantly contribute to melanoma progression. For instance, adipocytes transfer lipids directly to melanoma cells via the fatty acid transporter protein (FATP) family, particularly FATP1, which is overexpressed in melanoma. This lipid transfer enhances melanoma cell proliferation and invasion, contributing to a more aggressive tumor phenotype. Blocking FATP with inhibitors like Lipofermata has been shown to reduce lipid uptake in, the invasion of, and the growth of melanoma cells, demonstrating the potential of targeting adipocyte-melanoma interactions as a therapeutic strategy [116].

#### 2.5.3. Extracellular Matrix (ECM)

The ECM is a complex network of proteins and glycoproteins that provides structural support to tissues and influences cell behavior. In melanoma, the ECM plays a crucial role in regulating tumor growth, invasion, and metastasis.

##### Matrix Metalloproteinases (MMPs)

MMPs are enzymes that degrade various ECM components, facilitating melanoma cell invasion and metastasis. Elevated levels of MMPs, such as MMP-2 and MMP-9, are commonly observed in melanoma and are associated with aggressive tumor behavior [117,118]. MMPs break down collagen, laminin, and other ECM proteins, creating pathways for melanoma cells to invade surrounding tissues and spread to distant sites. This process is not solely driven by melanoma cells but also involves contributions from immune cells and cancer-associated fibroblasts (CAFs) within the tumor microenvironment.

Immune cells, including macrophages and neutrophils, can produce MMPs such as MMP-9, which further contributes to the degradation of ECM components and facilitates tumor progression. Macrophages can secrete MMPs in response to tumor-derived signals, exacerbating the invasive properties of melanoma [119,120]. Similarly, CAFs, which are abundant in the melanoma microenvironment, are significant producers of MMPs. These fibroblasts help to remodel the ECM, creating a more permissive environment for tumor growth and invasion. The interaction between melanoma cells and CAFs, along with the production of MMPs by these cells, plays a critical role in enhancing tumor invasiveness and metastasis [121].

MMP activity is tightly regulated by tissue inhibitors of metalloproteinases (TIMPs), and an imbalance between MMPs and TIMPs can enhance the invasive potential of melanoma cells [122]. Recent studies have highlighted the significant role of MMPs in melanoma progression. For example, a study analyzing the expression of MMPs in melanoma found that MMP-1, MMP-2, MMP-3, MMP-7, MMP-9, and MMP-13 showed increased levels in melanoma tissues compared to normal tissues. Specifically, MMP-9 expression was significantly higher in more aggressive melanoma subtypes, correlating with a 50% increase in metastatic potential [123].

Furthermore, another study demonstrated that the overexpression of MMP-2 and MMP-9 in melanoma cells is associated with increased tumor invasiveness and poor prognosis. The inhibition of these MMPs using specific inhibitors resulted in a substantial reduction in melanoma cell invasion and metastasis, highlighting their potential as therapeutic targets [120]. The balance between MMPs and TIMPs is crucial for maintaining tissue homeostasis. An imbalance, with elevated MMP activity and reduced TIMP levels, leads to increased ECM degradation and tumor progression. For instance, the study found that in melanoma with partial regression, MMP-1 and MMP-11 expressions were significantly lower in the regressed component compared to the non-regressed component, suggesting that MMP activity is closely linked to tumor aggressiveness and regression dynamics [120,123].

##### Integrins

Integrins are cell surface receptors that mediate cell–ECM interactions. They play crucial roles in melanoma cell migration, survival, and resistance to apoptosis. Integrins such as αvβ3 and α4β1 facilitate melanoma cell adhesion to ECM components like fibronectin, vitronectin, and laminin, promoting cell movement and invasion [124,125]. Targeting integrins with monoclonal antibodies or small-molecule inhibitors can disrupt these interactions, potentially inhibiting tumor progression and metastasis [126]. Recent studies have shown that blocking integrins like αvβ3 and αvβ5 can reduce melanoma aggressiveness by inhibiting pathways such as neuropilin 1 (NRP-1)-dependent angiogenesis. Inhibiting αvβ5 integrins, for instance, also blocks NRP-1, reducing VEGF-A mediated angiogenesis and tumor aggressiveness [127].

In addition, integrins such as α5β1 play a role in tumor angiogenesis. This integrin interacts with factors like angiopoietin 1 (ANG-1) and VEGF, promoting the formation of new blood vessels that supply the tumor with nutrients and oxygen, facilitating its growth and spread. Targeting α5β1 integrins can disrupt these processes, potentially limiting tumor growth and metastasis [128]. The diverse roles of integrins in melanoma highlight their potential as therapeutic targets. By developing monoclonal antibodies or small-molecule inhibitors that target specific integrins, it is possible to interfere with melanoma cell adhesion, migration, and invasion, ultimately improving patient outcomes.

#### 2.5.4. Soluble Factors

Soluble factors, including cytokines, chemokines, and growth factors, significantly shape the TME and promote melanoma growth and immune evasion.

##### Cytokines and Chemokines

These signaling molecules modulate inflammation, immune cell recruitment, and tumor cell behavior. Pro-inflammatory cytokines like IL-1, IL-6, and TNF-α, produced by various cell types including immune cells (e.g., macrophages and T cells), cancer-associated fibroblasts (CAFs), and tumor cells themselves, promote inflammation and tumor cell proliferation [129]. These cytokines can also induce the expression of adhesion molecules and chemokines that attract immune cells to the tumor niche [130,131]. In melanoma, elevated levels of IL-6 and TNF-α have been associated with increased tumor growth and poor prognosis. IL-6 has been shown to activate the JAK/STAT3 signaling pathway, leading to enhanced tumor cell survival and proliferation. Studies have demonstrated that high serum levels of IL-6 correlate with reduced overall survival in melanoma patients, highlighting its potential as a prognostic marker [132]. Chemokines such as CXCL8 (IL-8) promote cancer cell migration, invasion, and angiogenesis by interacting with their receptors on endothelial cells. Elevated levels of CXCL8 have been linked to increased melanoma aggressiveness and poor clinical outcomes. For example, a study found that high CXCL8 expression in melanoma tissues was associated with a 40% increase in tumor invasion and metastasis compared to tumors with low CXCL8 levels. Targeting CXCL8 or its receptors could, therefore, be a promising strategy to inhibit melanoma progression and improve patient outcomes [132].

##### Growth Factors

As mentioned briefly in previous sections, growth factors like VEGF, PDGF, and TGF-β, produced by various cell types including tumor cells, cancer-associated fibroblasts (CAFs), and endothelial cells, are crucial for angiogenesis, cell proliferation, and survival. Elevated levels of VEGF are associated with increased tumor angiogenesis and poor prognosis in melanoma patients. For instance, experimental studies have shown that combining VEGF inhibitors with other therapies can significantly enhance treatment efficacy. One study demonstrated that the combination of VEGF and TGF-β inhibitors led to a 27% rejection rate of B16 WT tumors, significantly improving the efficacy of anti-PD-1 and anti-CTLA-4 treatments by increasing the complete response rates by up to 80% in more immunogenic tumors [133,134]. TGF-β has a dual role in melanoma: it can suppress early tumor development by inhibiting cell proliferation but promotes advanced melanoma progression by enhancing immune evasion, metastasis, and ECM remodeling [135,136]. 

Moving beyond the tumor microenvironment, further critical aspects of melanoma biology are its heterogeneity and plasticity. These characteristics enable melanoma cells to adapt and survive under various conditions, contributing to treatment resistance and disease progression. Exploring the diverse and adaptable nature of melanoma cells sheds light on the challenges and potential strategies for effective therapy.

### 2.6. Heterogeneity and Plasticity

Melanoma is characterized by significant heterogeneity and plasticity, which contribute to its aggressive behavior and treatment resistance [137]. Tumor heterogeneity refers to the presence of diverse subpopulations of cancer cells within a single tumor, each with distinct genetic, epigenetic, and phenotypic profiles [138]. Plasticity is the ability of melanoma cells to dynamically change their phenotype in response to environmental signals and therapeutic pressures [139].

#### 2.6.1. Genetic Heterogeneity

Genetic heterogeneity in melanoma arises from mutations and genetic alterations that occur during tumor development and progression. These mutations can be clonal, present in all tumor cells, or subclonal, present only in a subset of cells [140]. Key genetic factors in melanoma are often distributed heterogeneously within tumors [141,142]. This genetic diversity leads to the coexistence of multiple subclones with different growth rates, metastatic potentials, and treatment responses.

##### Clonal Evolution

Tumors evolve through a process of clonal evolution, where genetic mutations accumulate over time, and subclones with advantageous traits are selected [143]. This process results in intratumoral heterogeneity, where different tumor regions harbor distinct genetic profiles. For instance, a study involving multiregion whole exome sequencing of metastatic melanoma revealed significant genetic heterogeneity within individual tumors, with different regions of the same tumor showing varied mutational landscapes. This intratumoral heterogeneity complicates treatment, as different subclones may respond differently to therapy [144].

##### Therapeutic Resistance

Resistance to treatments in melanoma can be classified into primary (intrinsic) and acquired (secondary) resistance. Primary resistance occurs when the tumor does not respond to treatment from the outset. This can be due to the genetic heterogeneity of the tumor, where cancer cells harbor genetic mutations that make them intrinsically resistant to drugs [145]. For example, some mutations in the NRAS gene can confer resistance to BRAF inhibitors, limiting the effectiveness of these treatments from the start. On the other hand, acquired resistance develops during treatment, often after an initial positive response [146]. Common mechanisms include the reactivation of the MAPK pathway, where secondary mutations in NRAS or MEK can reactivate the pathway even in the presence of BRAF or MEK inhibitors. Approximately 20% of acquired resistance cases are associated with this reactivation. Another mechanism is the activation of alternative pathways, such as the PI3K-AKT pathway. Mutations in PTEN or the overexpression of receptor tyrosine kinases (RTKs) like EGFR can activate this pathway, promoting cell survival and counteracting the effects of BRAF/MEK inhibitors [147,148,149,150]. Additionally, epigenetic modifications, including changes in DNA methylation and histone modifications, can alter gene expression and contribute to resistance [151]. 

Genetic heterogeneity contributes to therapeutic resistance by providing a reservoir of subclones that can survive initial treatment and repopulate the tumor. For example, subclones with mutations in genes that confer resistance to targeted therapies, such as BRAF inhibitors, can emerge and lead to tumor recurrence [152]. A recent study used dynamical modeling to explore proliferative–invasive plasticity and IFNγ signaling in melanoma, revealing mechanisms of PD-L1 expression heterogeneity. The study identified a minimal gene regulatory network (GRN) involving key players like MITF, SOX10, JUN, SOX9, and ZEB1. This network showed distinct phenotypic states (proliferative, invasive, neural crest-like) with varying PD-L1 levels and immune evasion traits. The study highlighted that PD-L1 levels can be dynamically regulated through cell state transitions, contributing to therapy resistance and immune evasion in melanoma [153].

#### 2.6.2. Phenotypic Plasticity

Phenotypic plasticity allows melanoma cells to switch between different cellular states in response to environmental signals and therapeutic pressures. This ability to dynamically alter phenotype is a key feature of melanoma and driven by both genetic and epigenetic mechanisms.

##### Epithelial–Mesenchymal Transition (EMT)

EMT is a process where epithelial cells acquire mesenchymal properties, increasing their migratory and invasive capacity. In melanoma, cells can undergo partial or complete EMT, enhancing their metastatic potential and treatment resistance [154,155,156]. For example, a study demonstrated that hypoxia induces a switch from a proliferative to an invasive phenotype in melanoma cells through the noncanonical Wnt5A signaling pathway, involving receptors ROR1 and ROR2. This switch significantly decreases sensitivity to BRAF inhibitors, leading to therapy resistance and highlighting the complexity of phenotypic plasticity in melanoma [157].

##### Stem Cell-like Properties

Some melanoma cells exhibit stem cell-like properties, characterized by the ability to self-renew and differentiate into various cell types. These melanoma stem cells are often more resistant to conventional therapies and can contribute to tumor relapse [158,159]. For instance, a recent study revealed that melanoma cells with high expression of the stem cell marker CD133 displayed greater resistance to chemotherapy and enhanced tumorigenic potential. These cells were also found to possess increased capabilities for invasion and metastasis, underscoring the role of stem cell-like properties in melanoma progression [160].

As we move forward, it is essential to delve deeper into other genetic markers that have not previously been mentioned. The following section will focus on additional defined or potential genetic markers currently under investigation. These markers offer further insights into the molecular mechanisms driving melanoma and hold promise for enhancing diagnosis, prognosis, and the development of targeted therapies.

## 3. Other Genetic Biomarkers

Genetic biomarkers are specific genes, mutations, or alterations that can be used to detect or predict the presence of cancer, determine prognosis, and guide therapeutic decisions. In melanoma, understanding these biomarkers is crucial for tailoring treatments to each patient, thereby improving outcomes and reducing unnecessary side effects.

Mutations in the *KIT* gene are particularly significant in specific melanoma subtypes, such as acral lentiginous melanomas and mucosal melanomas. These mutations activate the KIT tyrosine kinase receptor, driving tumor growth and survival. Tyrosine kinase inhibitors, such as imatinib, have shown efficacy in treating melanomas with activating *KIT* mutations, providing a tailored therapeutic approach for these patients [161,162]. In a study involving 90 patients with stage III or IV acral, mucosal, or cumulative sun-damaged skin melanoma, 11% of the melanomas tested had mutations in *KIT*. Among the patients treated with sunitinib, those with *KIT* mutations had complete remission in 15% of cases and partial responses in 30% of cases. This suggests that *KIT* mutations can be a significant marker for targeted therapy in melanoma [163,164,165].

Mutations in *GNAQ* and *GNA11* are predominantly found in uveal melanoma, a type of melanoma originating in the eye. These mutations activate G-protein signaling pathways, increasing cell growth and survival. Recent studies have identified potential therapeutic targets downstream of these mutations, such as protein kinase C (PKC) inhibitors. Darovasertib, a novel PKC inhibitor, has shown promise in preclinical studies, significantly decreasing cell viability in metastatic uveal melanoma. In a clinical study, patients treated with darovasertib demonstrated a 50% reduction in tumor size, highlighting the potential of targeting *GNAQ* and *GNA11* mutations to develop effective treatments for uveal melanoma [166,167,168,169].

In addition to these mutations, other genetic alterations serve as important prognostic biomarkers. For example, mutations in the tumor suppressor gene *TP53* are associated with poor prognosis in melanoma [170]. *TP53*, known as the “guardian of the genome,” regulates the cell cycle and prevents genomic instability [171]. When *TP53* is mutated, its ability to control cell division and apoptosis is compromised, leading to more aggressive tumor behavior [172]. Studies have shown that *TP53* mutations are found in approximately 20% of melanomas and are correlated with reduced overall survival and increased metastatic potential. UV radiation has been identified as a significant factor driving *TP53* mutations, with nearly 40% of UV-induced melanomas exhibiting *TP53* mutations [173,174].

Similarly, the loss of the tumor suppressor *PTEN*, which regulates the PI3K/Akt pathway, is associated with more aggressive melanoma and worse outcomes. Loss of *PTEN* results in uncontrolled cell proliferation and survival, contributing to tumor progression and treatment resistance [175,176]. Recent studies have demonstrated that *PTEN* loss co-occurs with *BRAF* mutations in 44% of BRAF mutant melanomas. This co-occurrence suggests that *PTEN* loss may contribute to the resistance to BRAF inhibitors, a common therapy for melanoma. The complete absence of the PTEN protein is associated with reduced overall survival in BRAF-mutant patients, further emphasizing its role in prognosis [177]. Furthermore, *PTEN* loss is linked to immune evasion mechanisms. It has been shown to mediate immune evasion by mitigating tumor antigen cross-presentation, resulting in T-cell exclusion. Tumors with *PTEN* loss often exhibit a non-T-cell inflamed phenotype, making them less responsive to immune checkpoint blockade therapies. In a cohort study, advanced-stage melanomas with *PTEN* loss were significantly associated with poorer survival outcomes compared to PTEN-positive tumors [178].

Predictive biomarkers are also crucial for determining which patients will benefit from specific treatments. For example, the expression of PD-L1 is a predictive factor for the response to immune checkpoint inhibitors. Tumors with high PD-L1 expression are more likely to respond to therapies that block the interaction between PD-1 on T cells and PD-L1 on tumor cells, thereby reactivating the immune response against the tumor [179,180]. A large meta-analysis found that patients with higher PD-L1 expression had significantly better responses to immune checkpoint inhibitors (ICIs). For example, in melanoma patients treated with anti-PD-1 or anti-PD-L1 therapies, those with PD-L1 expression levels greater than 1% showed response rates of approximately 45–50%, compared to 10–15% in patients with low or no PD-L1 expression [181]. Another study highlighted that patients with positive PD-L1 expression had higher overall survival rates. In a cohort of 210 advanced melanoma patients, those with positive PD-L1 expression (>1%) and *BRAF* V600 mutations demonstrated significantly higher survival rates after ICI therapy compared to patients without these biomarkers. Specifically, the study reported that PD-L1-positive patients had an overall survival benefit, suggesting that PD-L1 testing should be routinely assessed to identify patients most likely to benefit from ICI treatments [182]. Despite its importance, the predictive value of PD-L1 expression can vary. Factors such as the type of tissue tested, the specific PD-L1 assay used, and the cutoff values for PD-L1 positivity can influence the outcomes. For example, a review of FDA-approved ICIs indicated that PD-L1 expression predicted increased response rates in less than 30% of studies, with significant variability depending on the context and methodology used [183].

Emerging genetic biomarkers continue to refine the diagnosis and treatment of melanoma. Mutations in the promoter region of the *TERT* gene, which encodes the telomerase reverse transcriptase, are common in melanoma and associated with increased telomerase activity, promoting cellular immortality [184,185]. *TERT* promoter mutations are present in about 43% of sequenced melanoma samples. These mutations occur more frequently in non-acral cutaneous melanomas (48%) and melanomas with an occult primary (50%). They are less common in mucosal melanomas (23%) and acral melanomas (19%). Patients with *TERT* promoter mutations generally exhibit worse overall survival. For instance, in non-acral cutaneous melanoma patients, those with *TERT* promoter mutations had a median overall survival of 80 months compared to 291 months for those without the mutations [178]. In addition, the presence of *TERT* promoter mutations is often associated with co-occurring mutations in *BRAF* or *NRAS*, indicating a synergistic effect on melanoma progression. The mutations lead to increased *TERT* expression, resulting in enhanced telomerase activity, which in turn promotes tumor growth and cellular immortality. Interestingly, reverting these mutations has shown promise in preclinical studies. For example, reverting the *TERT* promoter mutation -146 C > T in melanoma cells led to significant growth inhibition both in vitro and in vivo, suggesting potential therapeutic strategies for targeting these mutations [186,187].

After examining various genetic markers that influence melanoma progression and treatment response, we turn our attention to immunotherapy. This groundbreaking approach harnesses the body’s immune system to recognize and combat melanoma cells, offering new hope for patients and expanding the arsenal of therapeutic strategies. The success and challenges of immunotherapy underscore its potential and complexity in the fight against melanoma.

## 4. Immunotherapy

Throughout this review, several currently approved therapies for treating melanoma skin cancer have been mentioned. Additionally, conventional therapies like surgical tumor removal as a first option, chemotherapy, and radiotherapy are still used in certain cases, although with more limited success. However, one of the most groundbreaking advances in melanoma treatment has been the development of immunotherapy, which harnesses the power of the immune system to fight cancer. Immunotherapy includes various approaches, but two of the most important are immune checkpoint inhibitors and vaccines.

### 4.1. Immune Checkpoint Inhibitors

Immune checkpoint inhibitors have transformed melanoma treatment by enhancing the body’s immune response against cancer cells. The significance of this advancement was highlighted when James P. Allison and Tasuku Honjo received the 2018 Nobel Prize in Physiology or Medicine for their discovery of cancer therapy based on the inhibition of negative immune regulation [188]. Normally, immune checkpoints are crucial for maintaining self-tolerance and modulating the duration and amplitude of physiological immune responses in peripheral tissues to minimize collateral tissue damage. However, melanoma cells can exploit these checkpoints to evade immune detection. Checkpoint inhibitors block these pathways, thereby reinvigorating T cells and enabling a robust antitumor response [189].

#### 4.1.1. PD-1/PD-L1 Inhibitors

**Figure 3:**
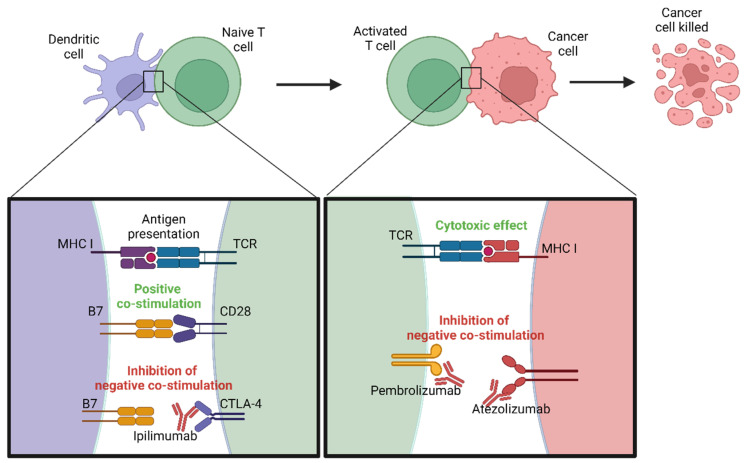
Illustration of Immune Checkpoint Pathways and their Role in Immunotherapy. The left panel shows the positive costimulation of T cells through the interaction of CD28 with B7 while simultaneously inhibiting the action of CTLA-4 thanks to blocking by ipilimumab. The right panel demonstrates the inhibition of negative costimulation by immune checkpoint inhibitors, such as pembrolizumab and ipilimumab, which block both PD-1 and PD-L1, leading to the enhanced cytotoxic effects of T cells against tumor cells. Created with BioRender.com.

Programmed cell death protein 1 (PD-1) is an inhibitory receptor primarily expressed on T cells. Its ligands, PD-L1 and PD-L2, can be expressed on tumor cells and other cells in the tumor microenvironment [190]. The interaction between PD-1 and PD-L1/PD-L2 inhibits T-cell activity, allowing tumors to escape immune surveillance. The mechanisms of action of some inhibitors, such as pembrolizumab (Keytruda) and nivolumab (Opdivo), block PD-1, as shown in Figure 3, preventing it from binding to its ligands and, thus, restoring T cell activity against melanoma cells [191]. Additionally, PD-L1 inhibitors like atezolizumab (Tecentriq) and durvalumab (Imfinzi) directly target PD-L1 on tumor cells, blocking its interaction with PD-1 and enhancing the immune response [192]. Clinical trials have demonstrated significant improvements in overall survival and progression-free survival with these agents, making them standard treatments for advanced melanoma [193]. A recent long-term study by Wolchok et al. demonstrated the sustained efficacy of immunotherapy in melanoma patients. The study reported a high response rate and durable survival in patients treated with anti-PD1, as well as those receiving a combination of anti-PD1 and anti-CTLA4. Notably, patients with *BRAF* V600E mutations exhibited better response rates compared to other subgroups, suggesting a significant clinical benefit of these therapies in this specific molecular context. These findings underscore the importance of immunotherapy as a key therapeutic strategy in melanoma management, particularly in patients with genetic characteristics that predispose them to a higher treatment response [194].

#### 4.1.2. CTLA-4 Inhibitors

Cytotoxic T-lymphocyte-associated protein 4 (CTLA-4) is another inhibitory receptor that downregulates immune responses. CTLA-4 is expressed on T cells and competes with CD28 for binding to B7 ligands (CD80/CD86) on antigen-presenting cells (APCs) [195]. While CD28-B7 binding provides an essential positive costimulatory signal for T-cell activation, CTLA-4-B7 binding delivers an inhibitory signal that dampens T-cell responses [196]. Ipilimumab (Yervoy) is an antibody targeting CTLA-4, preventing its binding to B7 ligands and, therefore, enhancing T-cell activation and proliferation (Figure 3). Although associated with significant immune-related adverse events, ipilimumab has shown durable responses and improved survival in some patients with advanced melanoma [87,197].

### 4.2. mRNA Vaccines

The goal of cancer vaccines is to stimulate the immune system to recognize and destroy cancer cells by presenting specific tumor antigens [198]. Unlike preventive vaccines, cancer vaccines are therapeutic and designed to treat existing cancers by enhancing the body’s immune response against the tumor. One of the most promising approaches in this field is the use of mRNA vaccines, especially those targeting neoantigens [199].

mRNA vaccines work by delivering genetic instructions to cells, allowing them to produce specific tumor antigens. These antigens are then presented on the cell surface, eliciting an immune response [198,199]. Neoantigens, which are tumor-specific antigens derived from mutations unique to cancer cells, are ideal targets for mRNA vaccines because they are highly immunogenic and not present in normal tissues. By targeting neoantigens, mRNA vaccines can effectively stimulate a robust and specific antitumor immune response [200].

**Figure 4:**
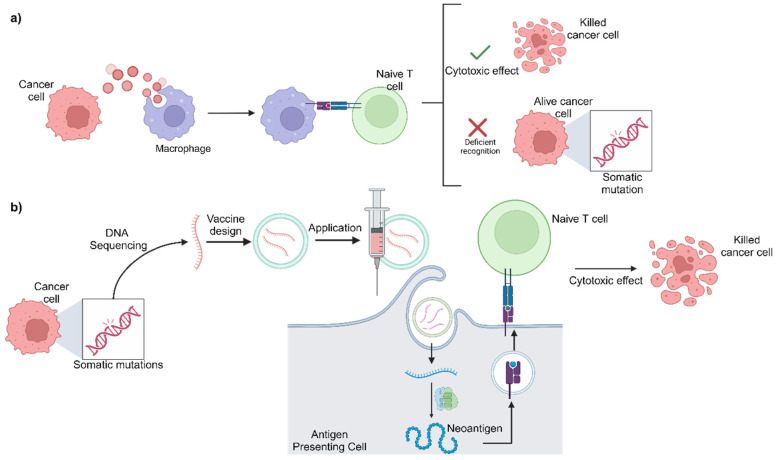
mRNA Vaccines. (**a**) Immune response against tumor cells: Macrophages are capable of phagocytosing and processing cells and molecules from the tumor niche to present antigens to T lymphocytes. These T cells identify the antigen presented on the surface of cancer cells and eliminate them. However, tumor cells carrying somatic mutations can produce neoantigens, thereby decreasing the efficiency of antigen recognition and the immune response. (**b**) Overview of the mechanism of mRNA vaccines targeting, melanoma: The process begins with the extraction of specific tumor antigens, which are then encoded into mRNA. The mRNA is encapsulated and administered to the patient, leading to the production of neoantigens by antigen-presenting cells (APCs). This stimulates an immune response, enhancing the cytotoxic activity of T cells against melanoma cells. Created with BioRender.com.

As shown in Figure 4, personalized mRNA vaccines are developed by sequencing the patient’s tumor to identify unique neoantigens. The mRNA encoding these neoantigens is then synthesized and administered to the patient. Moreover, combining mRNA vaccines with immune checkpoint inhibitors has been shown to enhance therapeutic efficacy. Checkpoint inhibitors, such as pembrolizumab and nivolumab, can relieve the immune suppression exerted by the tumor microenvironment, allowing T cells activated by the mRNA vaccine to function more effectively [201,202,203]. 

Recent clinical trials have demonstrated the potential of personalized mRNA vaccines in melanoma treatment. The phase IIb KEYNOTE-942 trial investigated the efficacy of the personalized mRNA-4157/V940 vaccine combined with pembrolizumab (Keytruda) in patients with high-risk melanoma following complete resection. The results showed that combination therapy significantly reduced the risk of disease recurrence by 44% compared to pembrolizumab alone. This trial involved 157 patients, and the combination therapy not only improved recurrence-free survival but also showed a favorable safety profile, with most side effects being mild and no severe immune-mediated toxicities being reported [204,205].

Building on the advancements in immunotherapy, we now shift our focus to emerging technologies that are revolutionizing melanoma research and treatment. These innovative approaches, including single-cell sequencing, CRISPR-Cas9 genetic editing, and AI-driven diagnostics, offer unprecedented precision and personalization in understanding and combating melanoma. These technologies are paving the way for new therapeutic strategies and improved patient outcomes.

## 5. Emerging Technologies

Single-cell sequencing technology has revolutionized our understanding of tumor heterogeneity and the tumor microenvironment. By analyzing the genetic and transcriptomic profiles of individual cells within a tumor, researchers can identify diverse cell populations and uncover mechanisms of drug resistance. This high-resolution approach enables the development of more precise and personalized treatment strategies for melanoma [206,207,208]. Recent advancements in single-cell RNA sequencing (scRNA-seq) have further illuminated the tumor immune microenvironment, revealing distinct populations of immune cells, including cytotoxic CD8+ T cells and regulatory T cells, which play critical roles in tumor progression and responses to therapy. Studies have shown that single-cell sequencing can uncover heterogeneity among dendritic cells within tumors, which is crucial for understanding tumor immunity and developing new immunotherapeutic strategies [209,210]. However, the use of such technology faces challenges such as high cost, technical complexity, and the need for specialized bioinformatics tools to handle and interpret the vast amounts of data generated. Additionally, single-cell sequencing often requires fresh or well-preserved tissue samples, which may not always be available in clinical settings [211,212].

Similarly, CRISPR-Cas9 genetic editing is a powerful tool that allows researchers to modify specific genes within melanoma cells. This technology can be used to study the functions of genetic alterations and identify potential therapeutic targets. Additionally, CRISPR-based therapies offer the possibility of correcting genetic mutations in melanoma patients, paving the way for new gene therapy approaches [213,214]. For instance, an analysis of CRISPR-Cas9 screens identified genetic dependencies in melanoma by comparing data from 28 melanoma cell lines and 313 cell lines of other tumor types. This study found an average of 1,494 fitness genes in each melanoma cell line and identified 33 genes whose inactivation specifically reduced the fitness of melanoma cells. Notably, the inactivation of DUSP4 and PPP2R2A significantly reduced melanoma cell proliferation. DUSP4 encodes an inhibitor of ERK, suggesting that further activation of MAPK signaling activity through its loss is selectively deleterious to melanoma cells [213]. Despite its potential, CRISPR technology also has several limitations, including off-target effects, which can lead to unintended genetic alterations. Moreover, the efficient and targeted delivery of CRISPR components to melanoma cells remains a significant challenge. There are also significant ethical concerns regarding the use of CRISPR, particularly in the context of human germline editing, which could have irreversible effects on future generations. These bioethical considerations necessitate stringent regulatory oversight and thoughtful deliberation about the long-term implications of gene editing technologies [215,216,217]. 

Artificial intelligence (AI) and machine learning (ML) are increasingly being applied to cancer research and treatment. These technologies can analyze large datasets from genomic, imaging, and clinical studies to identify patterns and predict treatment outcomes. AI algorithms enhance diagnostic accuracy by improving the interpretation of dermatoscopic images and detecting early signs of melanoma. Furthermore, ML models can predict patient responses to therapies, helping to tailor treatments to individual patients [218,219,220,221]. A recent study compared the diagnostic accuracy of human experts and machine learning algorithms in classifying pigmented skin lesions. The study involved 511 human readers and 139 ML algorithms. The top three algorithms achieved a mean accuracy of 87.3%, outperforming the 27 dermatologists whose mean accuracy was 86.6%. The area under the receiver operating characteristic curve (AUC) for the best algorithm was 0.89, compared to 0.87 for the dermatologists, demonstrating the statistically significant superior performances of the AI algorithms in diagnosing pigmented skin lesions [222]. Nevertheless, and similarly to single-cell sequencing, the implementation of AI and ML in clinical practice is fraught with challenges, including the need for extensive training datasets, potential biases in algorithm development, and issues related to data privacy and security [223,224].

Liquid biopsies provide a minimally invasive method to detect and monitor melanoma. By analyzing circulating tumor DNA (ctDNA) and other biomarkers in blood samples, liquid biopsies offer real-time insights into tumor dynamics, detect minimal residual disease, and monitor treatment responses. This approach serves as an alternative to traditional tissue biopsies and allows for the continuous tracking of disease progression and therapeutic efficacy [225]. Recent advancements have highlighted the potential of liquid biopsies to significantly enhance melanoma care. For example, technologies such as the “CTC-Chip” have been developed to capture circulating tumor cells (CTCs) from blood samples, improving the detection and analysis of tumor-specific markers. These methods enable the detailed characterization of tumor subtypes and staging, facilitating personalized treatment plans [226]. However, liquid biopsies also face several challenges, such as the relatively low abundance of ctDNA in blood, the need for highly sensitive detection methods, and the current lack of standardized protocols for ctDNA analysis [227,228,229].

The potential of nanotechnology to enhance melanoma treatment is being explored. Nanoparticles can be designed to deliver drugs, gene therapies, or immunotherapies directly to tumor cells with high precision, reducing unwanted effects and improving therapeutic efficacy. Additionally, nanotechnology-based imaging agents can improve melanoma detection and visualization, aiding early diagnosis and surgical planning [230]. For instance, nanoparticles like liposomes, carbon nanotubes, metal-based nanoparticles, and polymeric nanocarriers have been utilized to enhance the delivery of chemotherapeutic agents, leading to higher therapeutic efficacy and reduced side effects. Additionally, nanotechnology-based imaging agents are improving melanoma detection and visualization, aiding early diagnosis and surgical planning. These advancements show promise in transforming the approach to melanoma treatment, providing more targeted and efficient therapeutic options [230,231,232]. However, the translation of nanotechnology from the laboratory to clinical practice is hindered by challenges such as potential toxicity, stability issues, and the need for rigorous regulatory approval processes [233,234,235].

Adoptive cell therapies, such as CAR-T cell therapy and TIL therapy, show promise in treating melanoma. CAR-T cell therapy involves engineering a patient’s T cells to express chimeric antigen receptors (CARs) that specifically target melanoma antigens. TIL therapy involves expanding tumor-infiltrating lymphocytes outside the body and reinfusing them into the patient. These approaches aim to enhance the body’s natural immune response against melanoma and have shown encouraging results in clinical trials [236,237,238]. Recent advancements in TIL therapy have led to FDA approval of lifileucel (Amtagvi) for patients with unresectable or metastatic melanoma who have progressed on or after standard treatments. In the C-144-01 study, lifileucel achieved an objective response rate (ORR) of 31.4% among 153 patients, with 8 complete responses and 40 partial responses. The median duration of response (DOR) was not reached at a median follow-up of 27.6 months, with 41.7% of the responses maintained for at least 18 months. The median overall survival (OS) was 13.9 months, and the median progression-free survival (PFS) rate was 4.1 months. These results demonstrate lifileucel’s potential to provide durable responses in a heavily pretreated patient population with limited treatment options [239,240]. Nonetheless, adoptive cell therapies are associated with significant challenges, including high costs, complex manufacturing processes, and potential severe side effects such as cytokine release syndrome and neurotoxicity [241,242,243].

## 6. Future Directions

The future of melanoma treatment lies in the integration of emerging technologies and the continued advancement of personalized medicine. Combining treatment modalities such as targeted therapies, immunotherapies, and epigenetic therapies holds promise for overcoming resistance and improving patient outcomes. Ongoing research aims to identify optimal combinations and sequences of treatment to maximize efficacy. Additionally, the discovery of new biomarkers for early detection, prognosis, and treatment response prediction is crucial for advancing melanoma treatment. This will enable more precise patient stratification and tailored therapies.

Developing highly personalized treatment plans based on the genetic and molecular profiles of individual tumors represents the next frontier in melanoma treatment. Precision medicine seeks to provide more effective and less toxic therapies, thereby improving patient outcomes and quality of life. Innovative clinical trials are essential for testing new drugs, combinations, and treatment strategies. These trials will accelerate the translation of research discoveries into clinical practice, offering new hope to melanoma patients.

## 7. Conclusions

Melanoma represents one of the most complex challenges in oncology due to its aggressiveness, propensity for metastasis, and remarkable resistance to various treatments. Throughout this review, we have explored the genetic landscape of melanoma, highlighting the importance of mutations in key genes such as BRAF, NRAS, and KIT, as well as the relevance of epigenetic modifications and interactions with the tumor microenvironment.

The tumor microenvironment (TME) plays a crucial role in melanoma progression and therapy resistance. Immune cells, such as tumor-associated macrophages (TAMs), regulatory T cells (Tregs), and myeloid-derived suppressor cells (MDSCs), contribute to an immunosuppressive milieu that enables tumor growth and protects melanoma cells from immune surveillance. For instance, TAMs can secrete factors that promote angiogenesis, tissue remodeling, and the suppression of cytotoxic T-cell activity. Tregs, on the other hand, inhibit the activation and proliferation of effector T cells, further dampening the antitumor immune response. Additionally, MDSCs are known to interfere with T-cell function and promote tumor progression through the release of immunosuppressive cytokines.

Stromal cells, including cancer-associated fibroblasts (CAFs), also significantly influence melanoma progression. CAFs can remodel the extracellular matrix (ECM), creating a physical barrier that impedes the infiltration of immune cells into the tumor. Moreover, CAFs produce various growth factors, chemokines, and cytokines that support tumor cell proliferation, invasion, and resistance to apoptosis. The crosstalk between melanoma cells and stromal components, thus, establishes a supportive niche that facilitates tumor survival and metastasis.

Soluble factors within the TME, such as cytokines, chemokines, and growth factors, further modulate the behavior of both tumor and immune cells. Pro-inflammatory cytokines like IL-6 and TNF-α can promote tumor cell proliferation and survival, while anti-inflammatory cytokines such as IL-10 contribute to immune evasion. Additionally, chemokines like CXCL12 play a role in recruiting immunosuppressive cells to the TME, enhancing the tumor’s ability to resist immune attack.

Emerging technologies, such as single-cell sequencing and CRISPR-Cas9 genetic editing, are revolutionizing our understanding of melanoma heterogeneity and plasticity. These tools allow for the more precise characterization of tumor subtypes and the identification of new biomarkers that can improve disease diagnosis and prognosis. Single-cell sequencing, in particular, has provided insights into the diverse populations of immune and stromal cells within the TME, revealing their distinct roles and interactions in promoting melanoma progression and therapy resistance.

Immunotherapy, especially immune checkpoint inhibitors and personalized mRNA vaccines targeting neoantigens, has drastically changed the approach to melanoma treatment. These therapies have demonstrated significant efficacy by enhancing the body’s immune response against tumor cells, improving patient survival and quality of life. However, the effectiveness of immunotherapy can be limited by the immunosuppressive TME. Understanding the dynamic interactions between immune cells, stromal cells, and soluble factors is essential for developing strategies to overcome resistance and enhance the efficacy of immunotherapeutic approaches.

Despite these advances, the emergence of resistance mechanisms remains a considerable obstacle. Combined therapies, including the combination of BRAF and MEK inhibitors, as well as the integration of new strategies such as nanomedicine and liquid biopsies, offer hope for overcoming these barriers and achieving more durable therapeutic responses. Targeting the TME components, such as TAMs, CAFs, and specific cytokines, represents a promising strategy to enhance the effectiveness of existing therapies and prevent resistance.

In the future, precision medicine, based on the detailed characterization of each tumor’s genetic and molecular profiles, will be crucial for developing personalized treatments. Ongoing research into biomarkers and conducting innovative clinical trials will be essential for accelerating the translation of these discoveries into clinical practice. A comprehensive understanding of the TME, along with the integration of advanced technologies and therapeutic strategies, will be pivotal for advancing melanoma treatment and improving patient outcomes.
